# Thermal efficiency of a thermocell made of Prussian blue analogues

**DOI:** 10.1038/s41598-018-33091-w

**Published:** 2018-10-03

**Authors:** Takayuki Shibata, Yuya Fukuzumi, Yutaka Moritomo

**Affiliations:** 1 National Institute of Technology, Gunma College, Maebashi, Gunma 371–8530 Japan; 2 Graduate School of Pure and Applied Sciences, University of Tsukuba, Tsukuba, 305–8571 Japan; 3 Faculty of Pure and Applied Sciences, University of Tsukuba, Tsukuba, 305–8571 Japan; 4 Tsukuba Research Center for Energy Materials Science (TREMS), University of Tsukuba, Tsukuba, 305–8571 Japan

## Abstract

Recently, it was reported that a thermocell can convert temperature into electric energy by using the difference in the thermal coefficient (*α* = d*V*/d*T*) of the redox potential (*V*) between the cathode and anode materials. Among battery materials, Prussian blue analogues (PBAs) are promising materials for thermocell, because *α* changes from approximately −0.3 mV/K in Na_*x*_Mn[Fe(CN)_6_]_0.83_ 3.5 H_2_O (NMF83) to approximately 1.3 mV/K in Na_*x*_Co[Fe(CN)_6_]_0.9_2,9H_2_O (NCF90). In this work, we systematically investigated the thermal efficiency (*η*) of the NMF83/NCF90 thermocell relative to the difference (Δ*T*) between low (*T*_L_ = 282 K) and high (*T*_H_ = 292–338 K) temperatures. We found that the thermal efficiency (*η*) increased proportionally with Δ*T*. The linear increase in *η* is ascribed to the linear increase in the cell voltage (*V*_cell_) and the charge (*Q*_NCF90_) extracted from NCF90. Moreover, *η* reached 3.19% at Δ*T* = 56 K, which corresponds to 19% of the Carnot efficiency (*η*_carnot_ = 17.0%). We further confirmed that the magnitude of *Q*_NCF90_ is quantitatively reproduced by the slopes of the discharge curves of NMF83 and NCF90.

## Introduction

A new thermoelectric technology, that converts waste heat near room temperature and/or human body heat to electric energy at low cost and high efficiency, is required for a “smart” society. A semiconductor-based thermoelectric device, that uses the so-called Seebeck effect, is a promising technology and is.applied for practical use in Peltier cooling and thermal power generation in space vehicles^1^. However, the current devices must be bulky and heavy to convert the temperature difference between the electrodes into a sufficient voltage, which is an inevitable disadvantage of the device.

**Figure 1:**
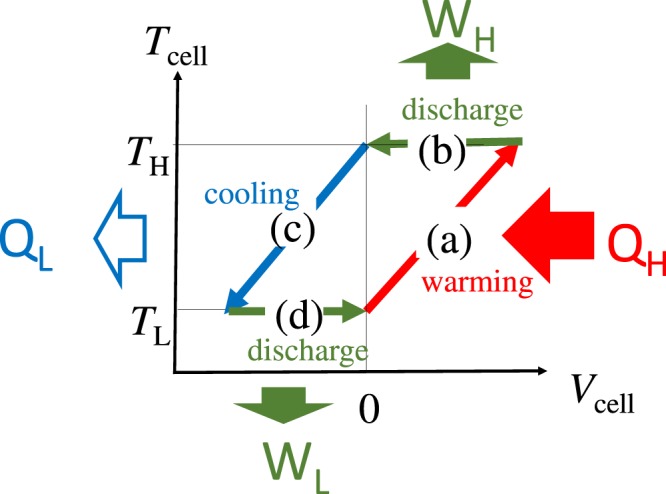
Schematic illustration of thermal cycle vs. voltage (*V*_cell_) and temperature (*T*_cell_) of the thermocell. The cycle consists of four processes. (**a**) Heating from *T*_L_ to *T*_H_, (**b**) discharge at *T*_H_, (**c**) cooling from *T*_H_ to *T*_L_, and (**d**) discharge at *T*_L_. Processes (**a**) and (**c**) are performed in the open circuit condition. *Q*_H_ and *Q*_L_ are the inflow and outflow of heat, respectively, and *W*_H_ and *W*_L_ are the electric works at *T*_H_ and *T*_L,_ respectively.

Recently, several researchers^2–5^ reported that a thermocell that uses the difference in the thermal coefficient (*α* = d*V*/d*T*) of the redox potential (*V*) between the anode (*α*_anode_) and cathode (*α*_cathode_) materials can convert the cell temperature (*T*_cell_) into the electric energy. The thermocell can produce electric energy in the thermal cycle between low (*T*_L_) and high (*T*_H_) temperatures, making in share contract with the semiconductor-based thermoelectric device. Figure 1 shows a schematic of stages of the thermocell thermal cycle: (a) warming from *T*_L_ to *T*_H_, (b) discharge at *T*_H_, (c) cooling from *T*_H_ to *T*_L_, and (d) discharge at *T*_L_. In the (a) warming process, the redox potentials of the anode and cathode change by *α*_anode_ and *α*_athode_, respectively. We expect a thermally induced change in *V*_cell_ as large as Δ*T*(*α*_cathode_ − *α*_anode_). In other words, electric energy is thermally stored in the thermocell. Some amount of the stored electric energy can be extracted by the (b) discharge process at *T*_H_. During the (b) cooling process, the redox potentials of the anode and cathode change by −*α*_anode_ Δ*T* and −*α*_cathode_ Δ*T*, respectively. The stored electric energy can be extracted by the (d) discharge process at *T*_L_. Lee *et al*.^2^ fabricated a thermocell with an anode and cathode made of [Fe(CN)_6_]^3+^/[Fe(CN)_6_]^4+^ ^6^ and a Prussian blue analogue (PBA) solid and succeeded in extracting electric energy. Yang *et al*.^3^. fabricated a thermocell with an anode and cathode made of Cu^+^/Cu^2+^ ^6^ and a PBA solid and succeeded in extracting electric energy. Shibata *et al*.^5^ fabricated a thermocell, consisting of two types of PBA solids with different *α* values. The thermocell produces electric energy with high thermal efficiency (*η* = 1%) between *T*_H_ (=295 K) and *T*_H_ (=323 K). This type of thermocell extends the application range of the battery materials from energy storage to energy conversion.

PBAs with chemical formulae are of Li_*x*_*M*[Fe(CN)_6_]_*y*_ and Na_*x*_*M*[Fe(CN)_6_]_*y*_ (*M* = transition metal) are promising candidates for cathode materials in lithium-ion and sodium-ion secondary batteries^7–16^. Most of the PBA materials have face-centrered cubic (fcc) (*Fm* $\bar{3}$ *m*; *Z* = 4) or trigonal (*R* $\bar{3}$ *m*; *Z* = 3) structures^17^, consisting of a three-dimensional (3D) jungle-gym-type host framework with guest Li^+^/Na^+^ ions and H_2_O molecules, which are accommodated in the nanopores of the framework. The framework contains considerable [Fe(CN)_6_] vacancies (10–30%). The discharge curves of Co- and Mn-PBAs show characteristic plateaus, the redox reaction of which are well assigned using X-ray absorption spectroscopy^18,19^. PBAs are also promising materials for thermocells because the magnitude and sign of *α* can be controlled by the chemical composition^20^. The *α* values of Na_*x*_Co[Fe(CN)_6_]_0.9_2,9H_2_O (NCF90) in the lower-lying plateau and the value of Na_*x*_Co[Fe(CN)_6_]_0.71_3.6H_2_O (NCF71) are ~1.3 and ~0.7 mV/K, respectively. Interestingly, the *α* of Na_*x*_Mn[Fe(CN)_6_]_0.83_ 3.5H_2_O (NMF83) in the lower-lying plateau is negative (approximately − 0.3 mV/K).

In our previous work^5^, we demonstrated that the NCF71/NCF90 thermocell can convert temperature into electric energy with 1% thermal efficiency between *T*_L_ = 295 K and *T*_H_ = 323 K. In this work, we systematically investigated the thermal efficiency (*η*) of the NMF83/NCF90 thermocell against the temperature difference (Δ*T*) between *T*_L_ and *T*_H_. The *η* of the NMF83/NCF90 thermocell is expected to be higher than that of the NCF71/NCF90 thermocell because Δ*α* (=*α*_cathode_ − *α*_anode_ ~ 1.7 mV/K) is much larger in the former cell. We found that *η* increases in proportion to Δ*T* and reaches 3.19% at Δ*T* = 56 K. The linear increase in *η* is ascribed to the linear increase in the cell voltage (*V*_cell_) and charge (*Q*_NCF90_) extracted from NCF90. We further confirmed that the magnitude of *Q*_NCF90_ is quantitatively reproduced by the slopes of the discharge curves in NMF83 and NCF90.

## Discharge curve of a half–cell

**Figure 2:**
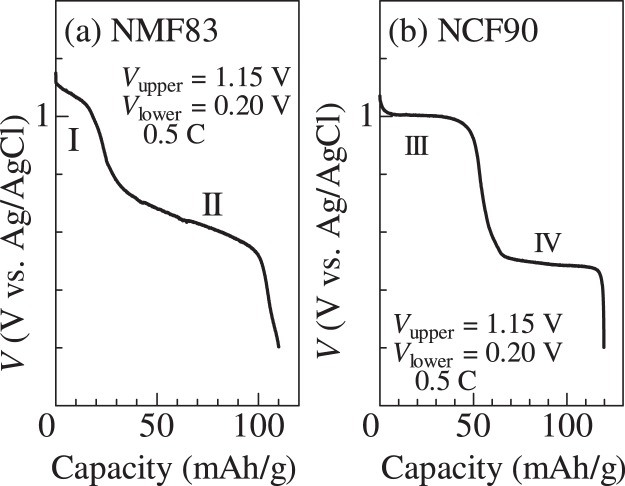
Discharge curves of (**a**) Na_*x*_Mn[Fe(CN)_6_]_0.83_ 3.5H_2_O (NMF83) and (**b**) Na_*x*_Co[Fe(CN)_6_]_0.9_2.9H_2_O (NCF90) films measured at 0.5 C. For convenience of explanation, we defined plateaus I, II, III, and IV.

Figure 2 shows the discharge curves of the (a) NMF83 and (b) NCF90 films. The curve of NMF83 [(a)] shows two plateaus (plateaus I and II) near 1.1 and 0.6 V vs. Ag/AgCl. Plateau I (<20 mAh/g) near 1.1 V is ascribed to the reaction^19^: Mn^3+^_0.49_Mn^2+^_0.51_[Fe^3+^(CN)_6_]_0.83_ + 0.49Na^+^ + 0.49 e^−^ → Na_0.49_Mn^2+^[Fe^3+^(CN)_6_]_0.83_. Plateau II (>20 mAh/g) near 0.6 V is ascribed to the reaction: Na_0.49_Mn^2+^[Fe^3+^(CN)_6_]_0.83_ + 0.83Na^+^ + 0.83 e^−^ → Na_1.32_Mn^2+^[Fe^2+^(CN)_6_]_0.83_. In the discharge process, Na^+^ ions are inserted into the framework, which causes reduction of Mn^3+^/Fe^3+^ to maintain the charge neutrality. The curve of NCF90 [(b)] shows two plateaus (plateaus III and VI) near 1.0 and 0.5 V vs. Ag/AgCl. Plateau III (<50 mAh/g) near 1.0 V is ascribed to the reaction:^18^ Co^3+^[Fe^3+^(CN)_6_]_0.6_[Fe^2+^(CN)_6_]_0.3_ + 0.6Na^+^ + 0.6 e^−^ → Na_0.6_Co^3+^[Fe^2+^(CN)_6_]_0.9_. Plateau VI (>50 mAh/g) near 0.5 V is ascribed to the reaction: Na_0.6_Co^3+^[Fe^2+^(CN)_6_]_0.9_ + Na^+^ + e^−^ → Na_1.6_Co^2+^[Fe^2+^(CN)_6_]_0.9_.

The difference (Δ*α* *=* *α*_cathode_ − *α*_anode_) in *α* is a crucial parameter for a thermocell, because *V*_cell_ increases in proportion to Δ*α*. Fukuzumi *et al*.^20^ systematically investigated the *α* values of prototypical PBAs against the Na concentration (*x*). In NMF83, *α* gradually decreases from 1.4 to − 0.4 mV/K with increasing in *x*. At plateau II, *α* becomes negative (approxmately −0.3 mV/K). In NCF90, *α* is ~0.4 mV/K and ~1.3 mV/K in plateau III and IV, respectively. Therefore, we can maximize Δ*α* if we chose NMF83 (plateau IV) and NCF90 (plateau II) as the anode and cathode, respectively. As discussed later, the slope of the plateau is another crucial parameter. In this work, we define the discharge curves of NMF83 [Fig. 1(a)] and NCF90 [Fig. 1(b)] as *V*_NMF83_(Q) and *V*_NCF90_(Q), respectively. The slopes, *i*.*e*., d*V*_NMF83_/d*Q* and d*V*_NCF90_/d*Q*, are roughly evaluated as −3.5 and −0.5 mV/(mAh/g) at plateaus II and IV, respectively.

## Thermal Cycle Measurement of Thermocell

We fabricated a two-pole beaker-type thermocell; for this thermocell, the anode, cathode and electrolyte are an as-grown NMF83 film, pre-oxidized NCF90 film, and an aqueous solutions containing 17 mol/kg NaClO_4_, respectively. The as-prepared thermocell was slowly cooled down to *T*_L_ (=282 K). At *T*_L_, the thermocell shows a finite *V*_cell_ (~0.1 V), which was discharged to 0 V under the constant current condition. If *T*_cell_ is slowly increased by Δ*T*(=*T*_H_ − *T*_L_) in the open circuit condition, the redox potentials of the anode and cathode change by *α*_anode_Δ*T* and *α*_cathode_Δ*T*, respectively, in the warming process. We expect a thermally induced change in *V*_cell_ as larger as Δ*T*(*α*_cathode_ − *α*_anode_). In other words, the electric energy is thermally stored in the thermocell. The amount of stored electric energy can be evaluated by the discharge process to 0 V under a constant current condition. The sweep area on the voltage-charge diagram corresponds to the stored electric energy. The behaviour of *V*_cell_ in the cooling process is opposite to that in the warming process. In this cooling process, the redox potentials of the anode and cathode change by −*α*_anode_Δ*T* and −*α*_cathode_Δ*T*, respectively. We expect a thermally induced change in *V*_cell_ as larger as −Δ*T*(*α*_cathode_ − *α*_anode_). The stored electric energy can be extracted by the discharge process to 0 V under constant current condition. Therefore, our thermocell can convert temperature to electric energy in the thermal cycle between *T*_L_ and *T*_H_.

**Figure 3:**
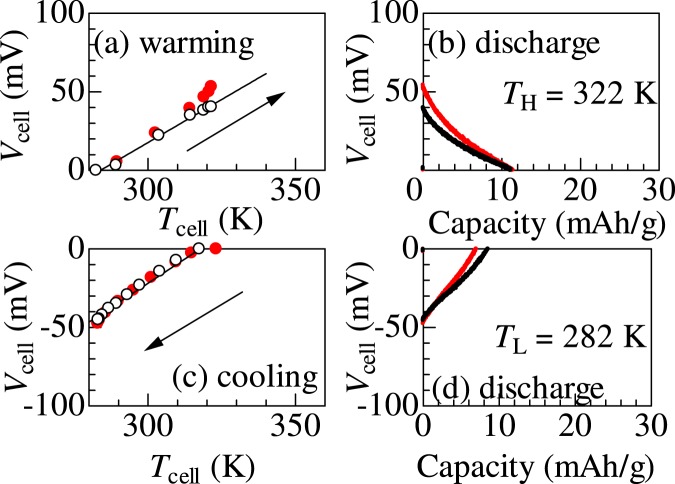
Temperature (*T*_cell_) and voltage (*V*_cell_) of the NCF83/NCF90 thermocell in the respective processes of the thermal cycle at Δ*T* = 40 K. (**a**) Warming process from *T*_L_ (=282 K) to *T*_H_ (=322 K) in the open circuit condition, (**b**) discharge process at *T*_H_ at constant current (*I* = 1.1 μA), (**c**) cooling process from *T*_H_ to *T*_L_ in the open circuit condition, and (**d**) discharge process at *T*_L_ at constant current (*I* = −1.1 μA). Black and red colours represent the data obtained in the first and second cycles, respectively.

Figure 3 shows a prototypical example of the thermal cycle of the NMF83/NCF90 thermocell at Δ*T* = 40 K. Black and red colours represent the data obtained in the first and second cycles, respectively. In the (a) warming process, *V*_cell_ linearly increases with the increase in *T*_cell_ at a rate of 1.2 mV/K. The rate is comparable to Δ*α* (~1.6 mV/K). At *T*_H_, the cell shows a finite voltage (*V*_cell_ = 40 mV), i.e., the cell is thermally-charged. In the (b) discharge process at *T*_H_, *V*_cell_ linearly decreases with the extracted charge. The final extracted charge (*Q*_NCF90_) from NCF90 is11.3 mAh/g, which is 10.8% of the discharge curve of NCF90 [Fig., 1(b)]. The electric work (*W*_H_ = 3.29 meV/NCF90) at *T*_H_ is roughly evaluated by *qV*_cell_/2, where *q* is the final extracted charge per NCF90. In the (c) cooling process, *V*_cell_ linearly decreases with the decrease in *T*_cell_ at a rate of 1.3 mV/K. At *T*_L_, the cell shows a finite voltage (*V*_cell_ = −44 mV). In the (d) discharge process, *Q*_NCF90_ was 8.4 mAh/g. The electric work (*W*_L_) at *T*_L_ is 2.82 meV/NCF90. The data in the second cycle are highly similar to those in the first cycle.

**Figure 4:**
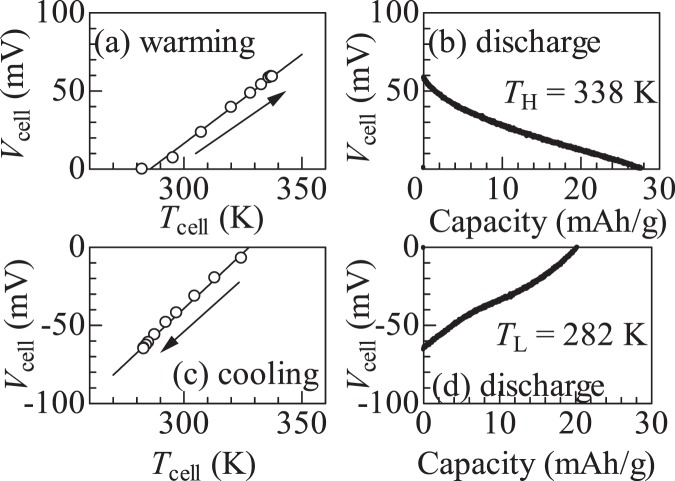
Thermal cycle (Δ*T* = 56 K) of the NCF83/NCF90 thermocell: (**a**) Warming process from *T*_L_ (=282 K) to *T*_H_ (=338 K) in the open circuit condition, (**b**) discharge process at *T*_H_ at constant current (*I* = 0.6 μA), (**c**) cooling process from *T*_H_ to *T*_L_ in the open circuit condition, and (**d**) discharge process at *T*_L_ at constant current (*I* = −0.6 μA).

Figure 4 shows another example of the thermal cycle at Δ*T* = 56 K. In the (a) warming process, *V*_cell_ linearly increases with the increase in *T*_cell_ at a rate of 1.1 mV/K. At *T*_H_ (=338 K), the cell shows a finite voltage (*V*_cell_ = 60 mV). In the (b) discharge process at *T*_H_, *V*_cell_ linearly decreases with the extracted charge, where *Q*_NCF90_ is 28.0 mAh/g, which is 26.7% of the discharge curve of NCF90 [Fig. 1(b)], and *W*_H_ is 12.80 meV/NCF90. In the (c) cooling process, *V*_cell_ linearly decreases with the decrease in *T*_cell_ at a rate of 1.2 mV/K. At *T*_L_ (=282 K), the cell shows a finite voltage (*V*_cell_ = −65 mV). In the (d) discharge process, *Q*_NCF90_ is 20.0 mAh/g and *W*_L_ is 9.70 meV/NCF90. Figure S1 shows the discharge curves at *T*_H_ and *T*_L_ against Δ*T*.

## Thermal Efficiency Against Δ*T*

The thermal efficiency (*η*) is defined by *W*_H_ + *W*_L_/*Q*_H_, where *Q*_H_ is the inflow of heat. Table 1 shows *W*_H_, *W*_L_, *Q*_H_, and *η* against Δ*T*. Furthermore, *W*_H_ (*W*_L_) was roughly evaluated as *qV*_cell_/2 at *T*_H_ (*T*_L_), where *q* is the final extracted charge per NCF90 in the discharge process at *T*_H_ (*T*_L_) and *Q*_H_ was evaluated as (*C*_anode_ + *C*_cathode_)Δ*T*, where *C*_anode_ (*C*_cathode_) is the heat capacity of anode (cathode) material. We neglected the heat capacity of the electrolyte because the amount of electrolyte is minimized in the current thermocell made of redox-capable solids. Using the specific heat (=4.16 meV/K per formula unit) of the ideal Na_2_Co[Fe(CN)_6_] in the Dulong-Petit law, *Q*_H_ is expressed as 4.16(1 + *n*)Δ*T* per NCF90, where *n* [=*n*_NMF83_/*n*_NCF90_. where *n*_NMF83_ (*n*_NCF90_) is the number of the the NMF83 (NCF90) units] is the molar ratio. Additionally, *W*_H_, *W*_L_, *Q*_H_, and *η* monotonously increases with Δ*T*, and *η* reaches 3.19% at Δ*T* = 56 K, which corresponds to19% of the Carnot efficiency (*η*_carnot_ = 17.0%).

**Table 1:** Parameters and performance of the NMF83/NCF90 thermocell.

Cycle	Δ*T* (K)	*W*_H_(meV/NCF90)	*W*_L_(meV/NCF90)	*d*_NCF90_ (mm)	*d*_NMF83_ (mm)	*n*	*Q*_H_(meV/NCF90)	*η* (%)
First	10	0.20	0.12	0.80	0.86	1.19	91.1	0.35
First	20	0.50	0.67	0.80	0.86	1.19	182.2	0.64
First	30	1.45	1.37	0.76	0.75	1.09	260.8	1.08
Second	30	0.98	1.23				260.8	0.85
First	39	3.07	2.58	0.42	0.49	1.27	368.3	1.53
Second	39	4.03	2.16				368.3	1.65
First	51	7.09	4.42	0.71	0.80	1.25	477.4	2.41
First	56	12.80	9.90	0.40	0.74	2.05	710.5	3.19

*n* [=*n*_NMF83_/*n*_NCF90_. where *n*_NMF83_ (*n*_NCF90_) is the number of NCF83 (NCF90) units] is the molar ratio. *d*_NCF90_ (*d*_NCF90_) is the thickness of the NMF83 (NCF90) film, and *W*_H_ (*W*_L_) and *Q*_H_ are the electric works at *T*_H_ (*T*_L_) and the inflow of heat, respectively.

## Comparison with Previous Works

In Table 2, we compare the cell parameters and *η* in the current thermocell with those in previously reported thermocells. In the thermocells reported in ref.^2^ and ref.^3^, ehe electrolyte is different between the anode and cathode and is separated by a membrane. However, the thermocells made of two types of PBA solids (ref.^5^ and this work) use the same electrolyte in the anode and cathode. As discussed in the following section, *η* increases linearly with Δ*T*. We should compare *η*/Δ*T*, rather than *η*, among the thermocells, and the *η*/Δ*T* values of the thermocells made of PBA solids are comparable to those of the thermocells reported in ref.^2^ and ref.^3^.

**Table 2:** Comparison of the parameters and thermal efficiency (*η*) of the thermocell.

Anode	Cathode	*α*_anode_ (mV/K)	*α*_cathode_ (mV/K)	*η* (%)	Δ*T* (K)	*η*/Δ*T* (%)	Ref.
CuHCF	Cu/Cu^2+^	−0.36	0.83	5.7	50	0.11	^2^
Fe(CN)_6_^3−^/Fe(CN)_6_^4−^	KFe[Fe(CN)_6_]	−1.46	0.00	2.0	40	0.05	^3^
NCF71	NCF90	0.53	1.32	1.0	28	0.04	^5^
NMF83	NCF90	−0.3^20)^	1.3^20)^	3.2	56	0.07	This work

Δ*T* is the difference between *T*_H_ and *T*_L_, and *α*_anode_ and *α*_cathode_ are the thermal coefficients of the redox potential of the anode and cathode, respectively.

In the thermocells made of PBA solids, *η*/Δ*T* (=0.07%/K) in the NMF83/NCF90 thermocell is much higher than that (=0.04%/K) in the NCF71/NCF90 thermocell. The enhancement of *η*/Δ*T* is ascribed to the larger Δ*α* in the NMF83/NCF90 thermocell. Specifically, Δ*α* is ~ 1.6 and 0.53 mV/K for the NMF83/NCF90 and NCF71/NCF90 thermocell, respectively.

## Discussion

**Figure 5:**
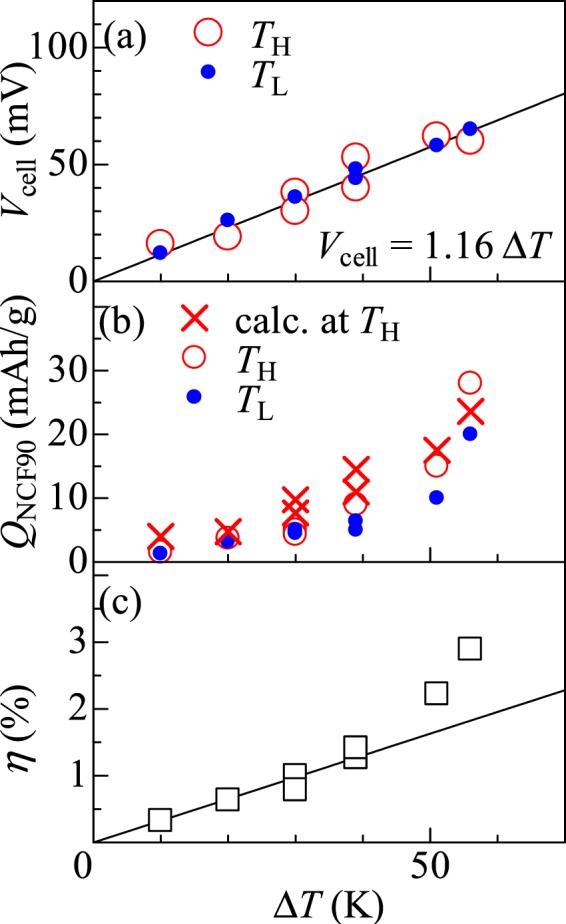
Δ*T* dependence of the (**a**) cell voltage (*V*_cell_), (**b**) final extracted charge (*Q*_NCF90_) from NCF90, and (**c**) thermal efficiency (*η*). The open and closed circles in (**a**) and (**b**) represent the data at *T*_H_ and *T*_L_, respectively. The solid lines in (**a**) and (**c**) indicate the results of least-squares fitting. The crosses in (**b**) are values calculated from the discharge curves of NMF83 and NCF90.

We turn the discussion to the Δ*T* dependence of *V*_cell_, *Q*_NCF90_, and *η*. Figure 5(a) shows the Δ*T* dependence of *V*_cell_. The open and closed circles represent the data at *T*_H_ and *T*_L_, respectively, and *V*_cell_ increases linearly with Δ*T*, as indicated by the solid line. The coefficient (=1.15 mV/K) is comparable to *α*_NCF90_ − *α*_NMF83_ (~ 1.6 mV/K). Thus, we experimentally confirm the relationship of *V*_cell_ = Δ*T*(*α*_NCF90_ − *α*_NMF83_).

Figure 5(b) shows the Δ*T* dependence of *Q*_NCF90_. The open and closed circles represent the data at *T*_H_ and *T*_L_, respectively. We quantitatively evaluate *Q*_NCF90_ at *T*_H_ from the discharge curves, *V*_NMF83_(Q) and *V*_NMF83_(Q), of NMF83 and NCF90. By applying the Tayloer expansion, *V*_cell_ at *T*_H_ is expressed as *Q*_NCF90_d*V*_NCF90_/d*Q* + (−*Q*_NNF83_d*V*_NMF83_/d*Q*), where *Q*_NCF90_ (*Q*_NMF83_) is the final extracted charges from NCF90 (NMF83). The first and second terms are negative, because d*V*_NCF90_/d*Q* < 0, d*V*_NMF83_/d*Q* < 0, and *Q*_NMF83_ < 0. The charge neutrality between the anode and cathode imposes a constraint condition of *m*_NMF83_*Q*_NMF83_ = −*m*_NCF90_*Q*_NCF90_, where *m*_NMF83_ (*m*_NCF90_) is the mass of NMF83 (NCF90). We obtaine *Q*_NCF90_ = −*V*_cell_/(d*V*_NCF90_/d*Q* + *m*^−1^d*V*_NMF83_/d*Q*), where *m* = *m*_NMF83_/*m*_NCF90_. We note that *m* = *n* (*M*_NMF83_/*M*_NCF90_), where *M*_NMF83_ (=338.5) and *M*_NCF90_ (=324.0) are the molecular weights of NMF83 and NCF90, respectively, and d*V*_NMF83_/d*Q* and d*V*_NCF90_/d*Q* are roughly evaluated as −3.5 and −0.5 mV/(mAh/g). In Fig. 5(b), these calculated *Q*_NCF90_ values at *T*_H_ are plotted with crosses. The calculated *Q*_NCF90_ satisfactorily reproduces the experimentally-obtained *Q*_NCF90_. We further confirmed the strong correlation between the calculated and experimentally-obtained *Q*_NCF90_ data at *T*_L_ (Fig. S2), thus, we demonstrating the empirical relationship of *Q*_NCF90_ = −*V*_cell_/(d*V*_NCF90_/d*Q* + *m*^−1^d*V*_NMF83_/d*Q*). These arguments clearly indicate that the slope of the discharge curve is a determining parameter of *Q*_NCF90_. In other words, the flatter the discharge curve becomes, the higher *η* becomes.

Figure 5(c) shows the Δ*T* dependence of *η*, where *η* linearly increase with Δ*T*, as indicated by the solid line., because *V*_cell_ [=Δ*T*(*α*_NCF90_ − *α*_NMF83_)], *Q*_NCF90_ [=−*V*_cell_/(d*V*_NCF90_/d*Q* + *m*^−1^d*V*_NMF83_/d*Q*)] and *Q*_H_ increases in proportion to Δ*T*.

## Summary

We systematically investigated *V*_cell_, *Q*_NCF90_, and *η* of the NMF83/NCF90 thermocell against Δ*T*. These three quantities increase linearly with Δ*T*, and *η* reaches 3.19% at Δ*T* = 56 K, which corresponds to 19% of the Carnot efficiency (*η*_carnot_ = 17.0%). We further confirmed that the magnitude of *Q*_NCF90_ is quantitatively reproduced by the slopes of the discharge curves of NMF83 and NCF90. These observations unambiguously illustrate us the strategy for enhancing *η* of the thermocell, that is, to explore and/or develop materials with a higher |*α*| and a flatter discharge curve.

## Method

### Fabrication and characterization of NMF83 and NCF90 films

Thin films of Na_*x*_Mn[Fe(CN)_6_]_0.83_ 3.5H_2_O (NMF83) and Na_*x*_Co[Fe(CN)_6_]_0.9_2,9H_2_O (NCF90) were synthesized by electrochemical deposition on an indium tin oxide (ITO) transparent electrode. Details of the synthesis conditions are described in literature^19,21^. The film area was 1.0 cm^2^. The NMF83 film consists of crystalline particles of a few hundred nm and the colour is white. The NCF90 film also consists of crystalline particles of a few hundred nm and the colour is light green. The film thicknesses were determined by a profilometer (BRUKER Dektak3030). The chemical composition of the film was determined by the inductively coupled plasma (ICP) method and CHN organic elemental analysis.

The synchrotron radiation X-ray powder diffraction (XRD) measurements were performed at BL02B2 beamline^22^ at the SPring-8. The films were removed from the ITO glass and were filled in 300 μm glass capillaries. The capillary was placed at the Debye Scherrer camera. The XRD patterns were monitored with a one-dimensional semiconductor detector (MYTHEN, Dectries Ltd.). The exposure time was 5 min. The wavelength of the X-rays (=0.69963 Å) was calibrated by the cell parameter of a standard CeO_2_ powder. Figure S3 shows the magnified diffraction pattern of NMF83 and NCF90. All reflections in NMF83 are assigned to a face-centrered cubic (fcc) structure (*Fm* $\bar{3}$ *m*; *Z* = 4), whereas those in NCF90 are assigned to a trigonal (hexagonal setting) structure (*R* $\bar{3}$ *m*; *Z* = 3). The cell parameters were refined using the Rietan-PF program^23^, and *a* = 10.5210(2) Å in NMF83, *a*_H_ = 7.4353(4) Å and *c*_H_ = 17.4758(11) Å in NCF90.

The charge/discharge curves of the NMF83 and NCF90 films were measured with a potentiostat (HokutoDENKO HJ1001SD8) using a three-pole beaker-type cell. The working, referential, and counter electrodes were the PBA film, a standard Ag/AgCl electrode, and Pt, respectively. The electrolytes consisted of aqueous solutions containing 17 mol/kg NaClO_4_. The charge/discharge rate was 0.5 C. The cut-off voltage was ranged from 0.20 to 1.15 V vs. Ag/AgCl. The mass of each film was evaluated using thickness, area, and density. We confirmed that the actual densities of the NMF83 and NCF90 films were 0.71 and 0.58 of the ideal density, respectively.

### Thermal cycle measurement of thermocell

The thermocell is a two-pole beaker-type cell (Fig. S4). The anode, cathode and electrolyte are the as-grown NMF83 film, pre-oxidized NCF90 film, and aqueous solutions containing 17 mol/kg NaClO_4_, respectively. Pre-oxidization of the NCF90 film was performed at *V*_upper_ = 0.65 V against Ag/AgCl in aqueous solutions containing 17 mol/kg NaClO_4_. The as-prepared thermocell was slowly cooled to *T*_L_ (=282 K). At *T*_L_, the thermocell show a finite *V*_cell_ (~0.1 V), which was discharged to 0 V under a constant current condition (0.1 C). The discharge rate was defined by the inverse of the charging time [hour] of the NCF90 film.

The thermal cycle measurement consists of four processes: (a) warming process from *T*_L_ to *T*_H_, (b) discharge process at *T*_H_, (c) cooling process from *T*_H_ to *T*_L_, and (d) discharge process at *T*_L_. In the (a) warming process, *T*_cell_ was slowly increased from *T*_L_ to *T*_H_ in the open circuit condition, and *T*_cell_ was monitored by a platinum resistance thermometer in the electrolyte. At (b) *T*_H_, the thermally-charged cell was discharged at 0.1 C. In the (c) cooling process, *T*_cell_ was slowly decreased from *T*_H_ to *T*_L_ in the open circuit condition. At (d) *T*_L_, the thermally-charged cell was discharged at 0.1 C. *T*_L_ was fixed at 282 K and *T*_H_ was changed from 292 K to 338 K.

## Electronic supplementary material

Supplementary Infomation
