# Supplementary material for: Thermal efficiency of a thermocell made of Prussian blue analogues
Source: Sci Rep. 2018 Oct 3;8:14784. doi: 10.1038/s41598-018-33091-w (PMC6170380; doi:10.1038/s41598-018-33091-w)
Supplement: Supplementary file 1 — Supplementary Infomation [file 41598_2018_33091_MOESM1_ESM.pdf]

Supporting information

Thermal efficiency of a thermocell made of Prussian blue analogues

Takayuki Shibata[1], Yuya Fukuzumi[2], and Yutaka Moritomo[2-4]*

[1]National Institute of Technology, Gunma College, Maebashi, Gunma 371-8530, Japan

[2]Graduate School of Pure and Applied Sciences, University of Tsukuba, Tsukuba 305-8571, Japan

[3]Faculty of Pure and Applied Sciences, University of Tsukuba, Tsukuba 305-8571, Japan

[4]Tsukuba Research Center for Energy Materials Science (TREMS), University of Tsukuba, Tsukuba 305-8571, Japan

Contact information:

Yutaka Moritomo

Graduate School of Pure and Applied Sciences

Faculty of Pure and Applied Sciences, and

Tsukuba Research Center for Energy Materials Science (TREMS)

Univ. of Tsukuba, Tennodai 1-1-1, Tsukuba 305-8571, Japan

Tel & Fax +81-29-853-4337

e-mail: moritomo.yutaka.gf@u.tsukuba.ac.jp

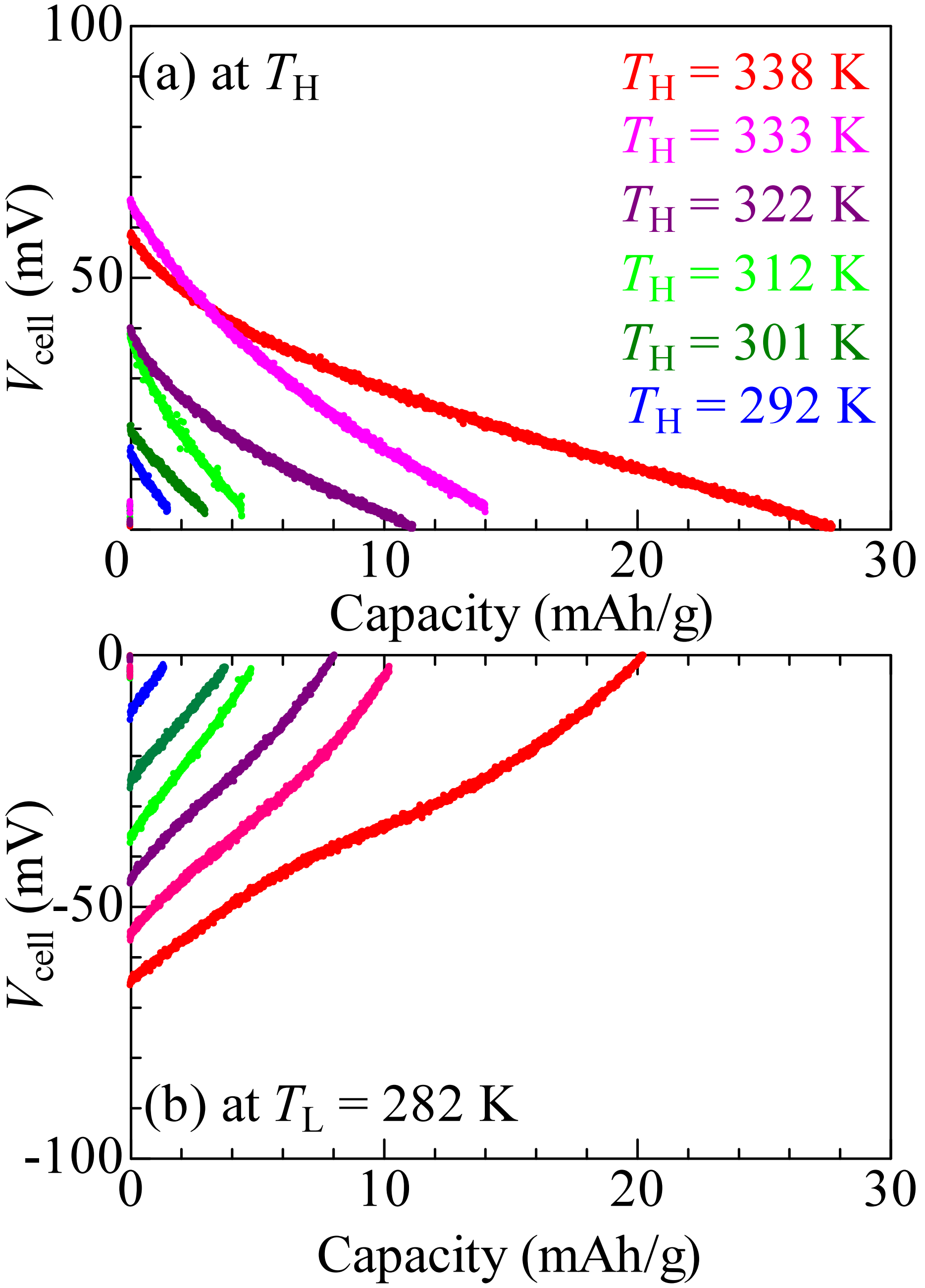

Fig. S1: Discharge curves at constant current (0.1 C) at (a) T_H and (b) T_L of the NMF83/NCF90 thermocell. T_L was fixed at 282 K.

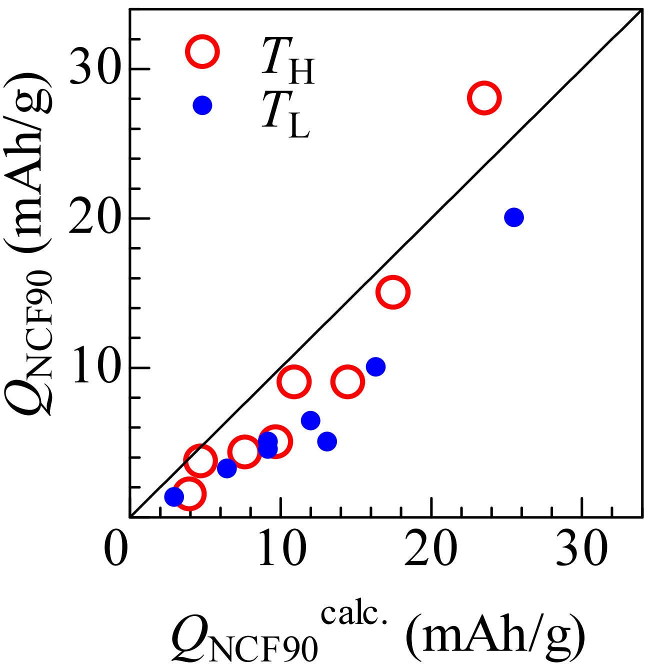

Fig. S2: Correlation diagram between experimental (Q_{NCF90}) and calculated ($Q_{NCF90}^{calc.}$) final extracted charge form NCF90 film in the discharge process. Open and closed circles represent the data at T_H and T_L, respectively.

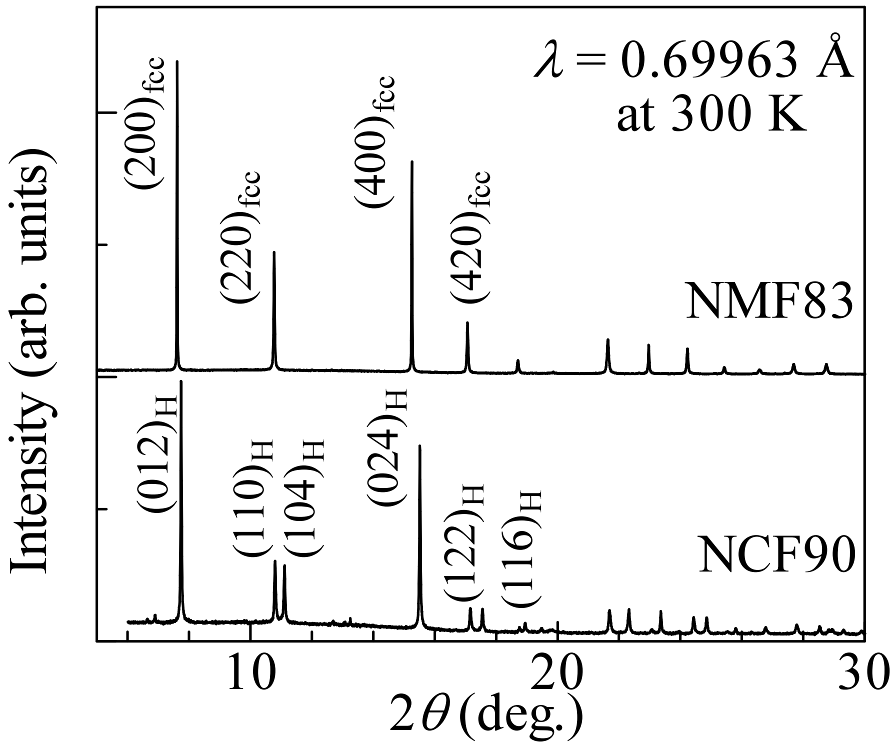

Fig. S3: X-ray powder diffraction patterns of as-grown NMF83 and NCF90. Values in parentheses represent indexes. The subscripts, fcc and H, represent face-centered cubic and trigonal (hexagonal setting) structures, respectively.

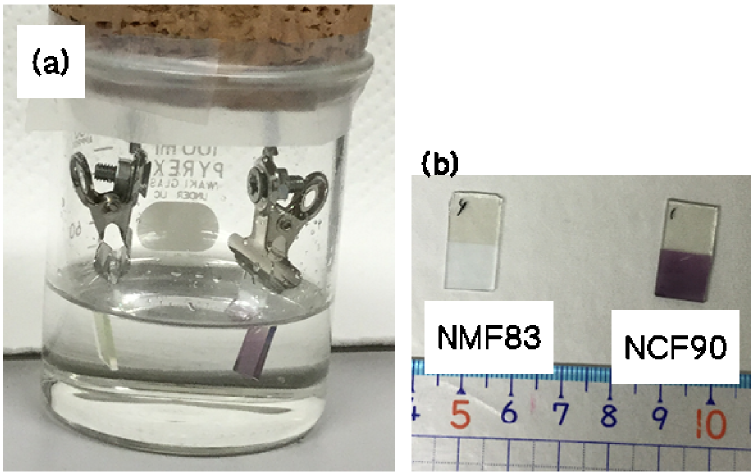

Fig. S4: (a) two-pole beaker-type cell. (b) as-grown NMF83 and pre-oxidized NCF90 films.
